# The effects of variable spatial aggregation on lymphatic filariasis transmission

**DOI:** 10.1186/s13071-024-06582-1

**Published:** 2025-01-09

**Authors:** Callum Shaw, Angus McLure, Kathryn Glass

**Affiliations:** National Centre for Epidemiology and Population Health, Australian National University, 62 Mills Road, Canberra, 2601 ACT Australia

**Keywords:** Lymphatic filariasis, Spatial heterogeneity, Individual heterogeneity, Model

## Abstract

**Background:**

Elimination of lymphatic filariasis (LF) is a World Health Organization goal, with several countries at or near prevalence thresholds. Where LF cases remain after mass drug administration, they tend to be spatially clustered, with an overdispersed individual worm burden. Both individual and spatial heterogeneities can cause aggregation of infection; however, few studies have investigated the drivers of heterogeneity and implications for disease elimination.

**Methods:**

We used a spatially explicit lymphatic filariasis model to investigate LF transmission in American Samoa at three spatial scales – a territory-level model, a village model with 64 groups and a subvillage model with 316 groups.

**Results:**

To reproduce American Samoan survey data, models with less spatial structure required increased individual-level bite aggregation. Threshold behaviour was present in the territory model but less evident in the models with spatial structure. As such, mass drug administration was most effective in the territory model, while in the spatially structured models, successive rounds of mass drug administration only gradually increased the likelihood of elimination. With the addition of spatial structure, residual infections remained in limited groups, and infection resurgence was slowed.

**Conclusions:**

Due to the impacts on potential intervention and surveillance strategies, it is critical that studies incorporate individual and spatial sources of heterogeneity to accurately model transmission and inform potential policy decisions.

**Graphical Abstract:**

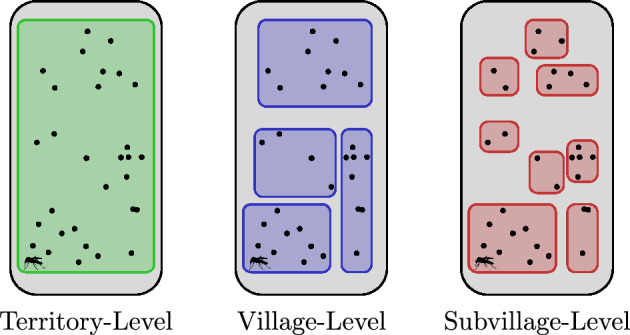

**Supplementary Information:**

The online version contains Supplementary Material available at 10.1186/s13071-024-06582-1.

## Background

Lymphatic filariasis (LF) is a preventable vector-borne helminth infection. While most LF-infected individuals are asymptomatic, chronic infections can result in long-term conditions such as lymphedema, elephantiasis and hydrocele. In 2000, the World Health Organization (WHO) launched the Global Programme to Eliminate Lymphatic Filariasis (GPELF). The programme aimed to eliminate LF as a public health problem by 2020 by interrupting transmission with mass drug administration (MDA) and to manage existing LF-related disability and morbidity. Under GPELF, there has been a 74% reduction in infections globally [1], and it has saved approximately 244 million disability-adjusted life years [2]. Despite substantial progress, by 2020, LF remained endemic in 72 countries, with 863 million people requiring treatment [3]. The WHO has released revised targets, which aim to have 58 of the remaining 72 endemic countries validated as having eliminated LF as a public health problem by 2030 [4].

Under GPELF, MDA initially involved several annual rounds of treatment of double-drug MDA, either diethylcarbamazine and albendazole or ivermectin and albendazole in areas where onchocerciasis is co-endemic. Since 2017, in areas without onchocerciasis, the WHO has recommended triple-drug MDA with ivermectin, diethylcarbamazine and albendazole (IDA) due to increased efficacy [5]. Once the required rounds of MDA with at least 65% coverage have been administered and sentinel and spot-check sites have found microfilariae (m.f.) prevalence is below 1%, transmission assessment surveys (TAS) are implemented. For bancroftian filariasis, TAS are deemed to have been passed if antigen prevalence is below 2% if *Anopheles* or *Culex* are the primary vectors or below 1% for *Aedes*-mediated transmission.

Current intervention strategies rely on the existence of critical prevalence thresholds. Below the prevalence threshold, it is believed that there is a low probability that any infected person would have both a sexually mature male and female worm, and therefore, most infections would be non-infectious and continued transmission is believed unsustainable [6]. Recently, there has been increased scrutiny on blanket target thresholds, as regions have passed TAS and had subsequent rebounds in transmission [7, 8]. Modelling studies have predicted that thresholds, if present, are likely highly dependent on local socio-ecological conditions [9–11]. Davis et al. [12] evaluated both modelling and experimental evidence and found blanket thresholds insufficient for determining elimination as a public health problem, especially in regions with high vector biting rates.

LF infection is aggregated, driven by both spatial- and individual-level heterogeneities. Strong spatial heterogeneity in transmission has been observed, even in geographically small regions, such as American Samoa [13], driven by vector abundance, biting rates [14] and human mixing [15, 16]. Individual-level heterogeneity is associated with factors such as age, sex, social and economic factors [17, 18]. Modelling studies have shown that individual-level heterogeneity varies by setting and can affect critical thresholds [10, 11]. Understanding the sources of heterogeneity is essential for analysing local transmission dynamics [19], particularly as modelling studies for other vector-borne diseases found movement [20, 21], heterogeneous exposure, mixing and limited population sizes [16, 22] all affect transmission.

In this study, we use a spatially explicit agent-based LF modelling framework to analyse the impacts of spatial structure on individual-level heterogeneity, critical threshold estimates, interventions and post-MDA surveillance efforts.

## Methods

We extended the existing GEOFIL model [23, 24] to include more realistic limitation and facilitation behaviour, variable infectivity of human hosts dependent on mature worm load and individual-level aggregation of mosquito bites. Previous iterations of GEOFIL included American Samoan residential buildings, schools and workplace locations and American Samoan-specific population dynamics. The model has been streamlined to be readily configured to other settings and is run on a network of interconnected groups. In this study, groups are defined as collections of residential buildings but could be any other natural population subgroup within which contact can be assumed to be well mixed.

### Variable grouping

**Fig. 1:**
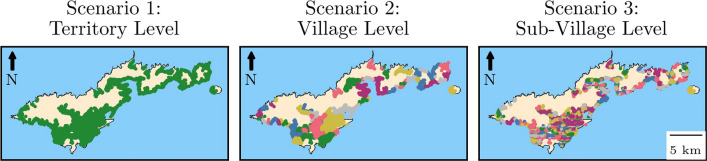
Three model scenarios, where each colour represents a group of residential buildings. Territory scenario: all residential buildings in the same group. Village scenario: residential buildings grouped into their respective villages. Subvillage scenario: residential buildings grouped on the basis of their proximity to other residential buildings

We modelled transmission in American Samoa at three different levels of spatial aggregation. Using over 13,000 previously surveyed residential building locations, we grouped buildings to form populations of varying sizes, connected by a commuting network. In the territory scenario, we removed all spatial components and modelled American Samoa as a single group. In the village scenario, we modelled transmission with 64 groups corresponding to American Samoan villages. In the subvillage scenario, 316 groups were created by aggregating residential buildings within a 25 m radius of each other. While there was no maximum group size, a group had to contain at least 20 residential buildings. If buildings were greater than 25 ms but less than 200 ms from their nearest neighbour, they were included in the closest group. The small number of residential buildings that were greater than 200 ms from the closest neighbouring building were excluded. A diagram of the three scenarios is given in Fig. 1, and the distribution of population group sizes for the two spatially explicit scenarios is given in Additional file 1, Supplementary Fig. 1. For clarity, the spatial scale of building aggregation included in each scenario will henceforth be referred to as spatial structure.

For each scenario, we assumed that contact was well mixed within each group. While there was no direct transmission between groups, commuting allowed individuals to belong to more than one group. In the village and subvillage scenarios, individuals were assigned a night-time and day-time group. The night-time group was their place of residence, while the day-time group was where the individual was located during working hours (i.e. home, school or workplace). When reporting the number and prevalence of infections in a group, only individuals in that group at night were included.

### Commuting network

**Fig. 2:**
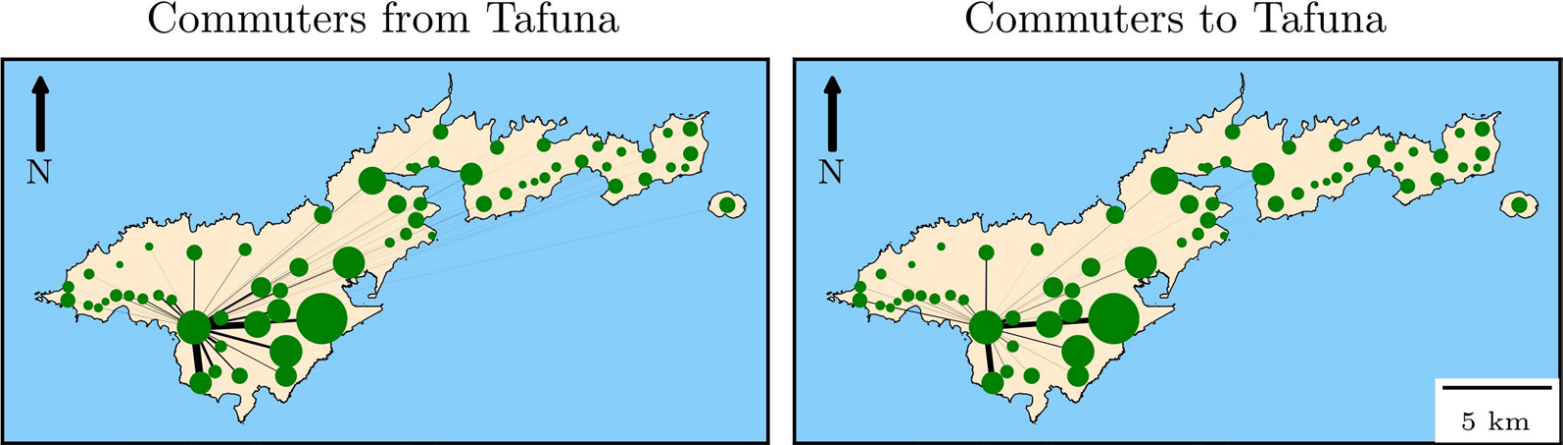
Example commuter networks in the village model for Tafuna. The green dots are village locations, and the dot size is proportional to population size. The black lines between villages represent commuter flux with line width proportional to flux magnitude. Commuter flux from Tafuna is given in the figure on the left, and commuter flux into Tafuna is given in the figure on the right

In the village and subvillage scenarios, the model used a commuting network to assign day-time groups. Only a proportion of individuals were assigned a commute, in line with Samoan labour force participation rates and commuting rates [25]. If an individual did not commute, their night-time and day-time groups were the same. We employed a radiation model [26] to predict commuting patterns between groups. The commuter flux from group *i* to *j* $T_{ij}$ was:

1 $$\begin{aligned} \langle T_{ij} \rangle = T_i\frac{m_i n_j}{(m_i+s_{ij})(m_i+n_j+s_{ij})}, \end{aligned}$$

where $m_i$ and $n_j$ are the total populations of group *i* and *j*, respectively. The distance between the centroids of the two groups is $r_{ij}$, and $s_{ij}$ is the total population within a radius $r_{ij}$ of group *i* excluding $m_i$ and $n_j$. The total population of commuters from group *i* ($T_i$) equals $\sum _{j \ne i} T_{ij}$. Commuting networks were fixed during the simulation period. Unlike previous iterations of GEOFIL, all individuals above the age of 5 years were included in the commuter network, as schools were no longer explicitly modelled. An example of the commuter flux to and from Tafuna, the most populous village in American Samoa, is given for the village scenario in Fig. 2.

### Transmission

Infection was modelled as a micro-simulation in the human agents, tracking worm age, gender, reproductive maturity and sterilisation status in infected individuals. An infectious bite was one that transmitted immature worms that survived until maturity, and individuals could receive infectious bites in both their day-time and night-time groups. The number of infectious bites an individual *i*, in group *j*, at period of the day *x*, received each time step was modelled as a Poisson random variable with rate ($\varvec{\lambda }$) equal to

2 $$\begin{aligned} \varvec{\lambda }_{i,j,x} = c_i b_{i} \phi _{x} \frac{\sum _{n\in N_{j,x}}c_n b_{n} f(W_n)}{ \sum _{k\in N_{j,x}} c_n b_{n}}, \end{aligned}$$

where $N_{j,x}$ is the set of all individuals in group *j* at period *x*, $c_i$ is the age-dependent relative biting exposure, $\phi _x$ is the proportion of bites that occur either during either day-time or night-time and $b_{i}$ is the individual’s relative infectious bite risk. This bite risk introduced individual-level heterogeneity; similar to Irvine et al. [11], it was modelled as a gamma random variable with shape parameter *k* and scale parameter 1/*k*. As the magnitude of aggregation is setting-specific, *k* was a fitted parameter. Further explanation on individual-level aggregation in the model is given in Additional file 1. We assumed that adult worms were polygamous and well mixed within a host. Therefore, for an infected individual, the number of mated female worms was assumed to equal the number of mature female worms if there was at least one mature male worm and zero otherwise. The variable, $W_i$ is the mated worm load of individual *i* adjusted for sterilising effects of MDA, while $f(W_i)$ accounted for density-dependent effects in the transmission process. Mosquito genera have been observed to have distinct relationships between m.f. uptake and larval yield. Limitation behaviour, where parasite yield saturates at high m.f. densities, has been observed in the *Aedes* genus [27–29]. As *Aedes* is the dominant vector in American Samoa, we assumed $f(W_i)$ took the following saturating form:

3 $$\begin{aligned} f(W_i) = A \theta _1 \left( 1- \textrm{e}^{-\theta _2 W_i}\right) , \end{aligned}$$

where $\theta _1$ is the base infectiousness and $\theta _2$ determines how rapidly infectiousness saturates with increasing mated mature worm load, and $A = 1 / (1 - \exp (-\theta _2))$ is used to normalise the saturation term so that $f(1) = \theta _1$ for all values of $\theta _2$.

Epidemiological processes (e.g. infection, worm maturation and worm death) were run on a 7-day time-step, while individual-specific processes (e.g. births and deaths) were run on a 28-day time-step.

### Model initialisation

The model was initialised with antigen and m.f. prevalence data from a 2010 community survey in American Samoa [30] that was conducted 4 years after the most recent round of MDA in 2006. Territory-wide antigen prevalence was set to 3.2% and m.f. prevalence to 0.5%. As information on the clustering of infections was not available in the 2010 survey, the village and subvillage models were initialised with m.f. intra-cluster correlation (ICC) values of 0.28 from the 2016 community survey [31]. ICCs were calculated by fitting a random effect logistic regression model and using the latent variable method. Further details of initialisation are given in Additional file 1.

### Model fitting

The three model scenarios were fit using approximate Bayesian computation. We used a simple rejection algorithm with a fixed tolerance and post-sampling adjustment. To account for inter-run variability, each sampled parameter set was run multiple times. The fitting used summary statistics from the results of community surveys conducted in 2010 and 2016 in American Samoa [30, 31] and used semi-informative uniform priors for model parameters. The scenarios were initialised in 2010 as described above and fitted to the 2016 survey results. The territory scenario, with no spatial structure, included two fitted parameters ($\theta _1$ and *k*), while the other scenarios included an additional fitted parameter, *w*, the ratio of transmission during the day-time to transmission at night-time. All scenarios were fitted to the observed 2016 m.f. and antigen prevalence. Village and subvillage scenarios were also fitted to the observed 2016 m.f. ICC results.

**Fig. 3:**
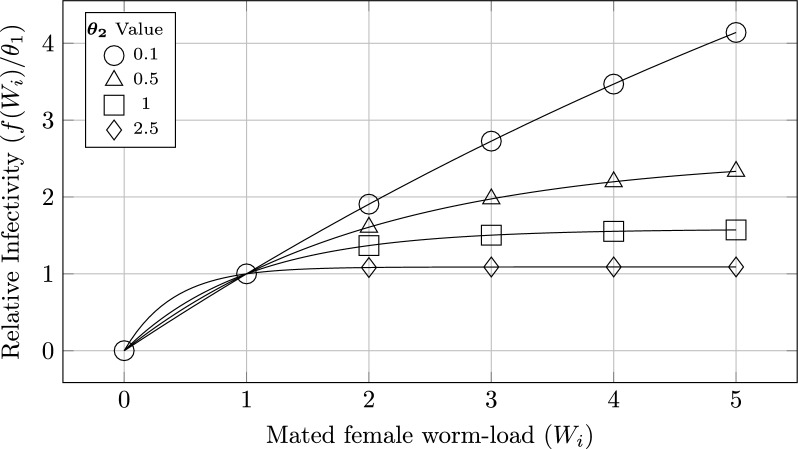
The relationship between mated worm load and an individual’s relative infectivity for four values of $\theta _2$

It is unknown how infectivity increases with the number of pairs of mature mated worms in the host. We modelled different limitation behaviours, parameterised by $\theta _2$ (Fig. 3).

### Interventions

We analysed the efficacy of territory MDA on LF transmission dynamics and critical threshold for each scenario. All interventions began in 2016 and involved $2-5$ annual rounds of triple-drug MDA. Following WHO recommendations, children aged under 2 years were excluded from MDA. The model assumed MDA coverage of 70%, with coverage calculated over the eligible population. Assumptions around MDA efficacy are given in Additional file 2, Supplementary Table 1.

LF was considered to be eliminated in a simulation if, at the end of the simulation period, there were either no m.f. positive people or the slope of a linear least-squares regression on the quarterly territory-level m.f. positive individuals count from 1-year post-MDA until the end of the simulation period was less than or equal to zero.

All simulations were run until 2031 from the medians of their fitted posteriors, and each configuration was run 500 times. To assess the sensitivity of intervention outcome to coverage, $2-5$ annual rounds of triple-drug MDA were simulated 100 times with 80% coverage.

## Results

### Fitting

Models with increased spatial structure had less individual-level bite aggregation and increased base infectiousness, suggesting a trade-off between spatial structure and individual-level bite aggregation (Additional file 3). Configurations with strong limitation behaviour (minimal increased infectivity with additional worms) had higher base infectiousness but little change in individual-level bite aggregation. Models with weak limitation behaviour produced unrealistic dynamics, particularly with the addition of spatial structure. In American Samoa, before the commencement of MDA in 2000, antigen prevalence was 16.5% [32]. Simulations with weak limitation behaviour ($\theta _2 = 0.1$) predicted antigen prevalence would reach approximately 30% by 2030 in the village and subvillage scenarios (Additional file 4, Supplementary Fig. 5) and 20% by 2030 with $\theta _2 = 0.5$. As fitted model dynamics were similar for the remaining values of $\theta _2$ (1 and 2.5), we focus on $\theta _2 = 1$ from here, with results for all values of $\theta _2$ given in Additional file 4.

### Intervention efficacy

**Fig. 4:**
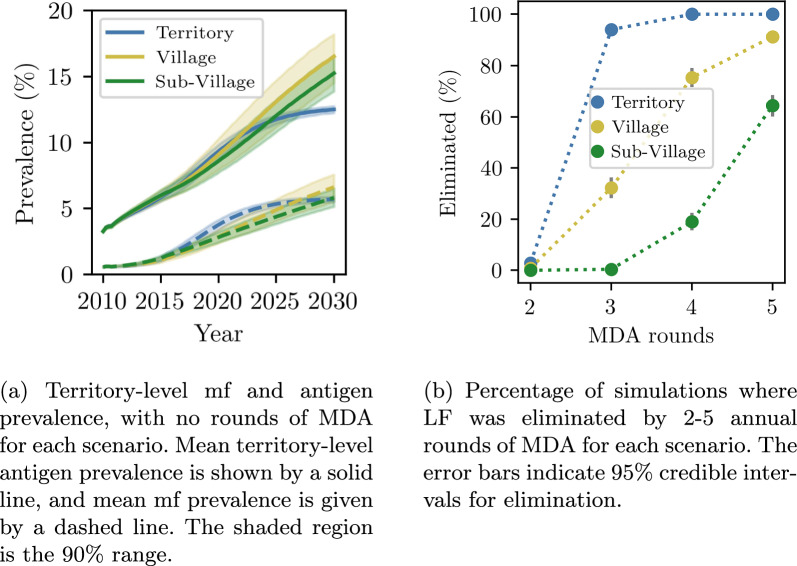
Territory-wide antigen and m.f. prevalence and MDA efficacy. In blue is the territory scenario, in gold is the village scenario and in green is the subvillage scenario

All model scenarios predicted that m.f. and antigen prevalence would continue to increase without future interventions (Fig. 4a). The village and subvillage scenarios predicted approximately linear growth in infections beyond 2016, while the territory scenario predicted that new infections would begin to level off after 2020, saturating by 2030.

All scenarios required two or more rounds of MDA to have a non-zero probability of elimination. Although the number of m.f.-positive individuals remaining immediately after MDA was similar across scenarios (Additional file 4, Supplementary Fig. 6), LF was less likely to be eliminated in scenarios with spatial structure (Fig. 4b). Elimination probability in the territory scenario rose sharply from 2.8% after two rounds of 70% coverage MDA to 94% after three rounds, with similar behaviour observed for 80% MDA coverage (Additional file 4, Supplementary Figs. 7 and 8). In contrast, the village and subvillage scenarios showed a gradual increase in the probability of elimination with successive rounds of MDA. To achieve elimination in a majority of simulations, the village and suvillage scenarios required four and five rounds of MDA, respectively.

### Critical thresholds

**Fig. 5:**
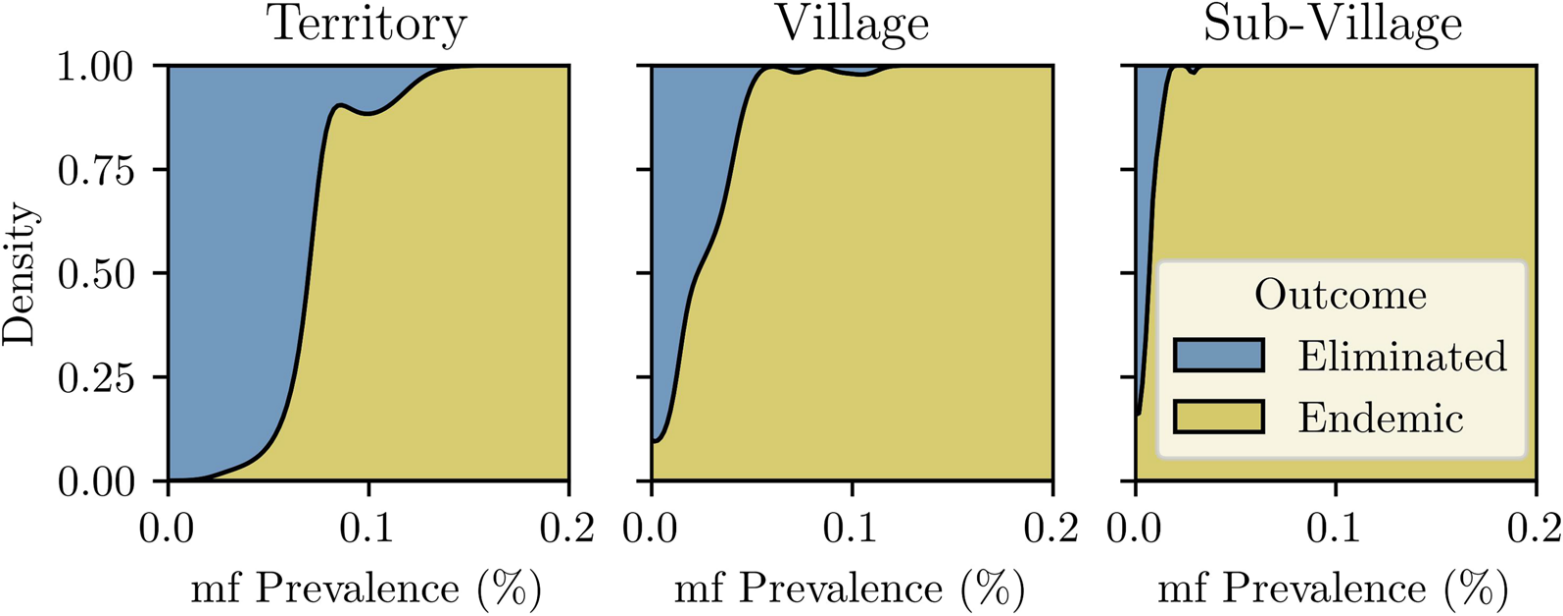
Probability of territory-wide elimination of LF, conditional on m.f. prevalence 1 year after the cessation of MDA. Simulations where LF remained endemic are in gold or was eliminated are in blue. Prevalence is calculated for the three scenarios at the territory level

Critical threshold behaviour changed with model specification (Fig. 5). The territory scenario displayed clear threshold behaviour, with elimination in > 75% of simulations if MDA reduced territory m.f. prevalence to less than 0.064%. In contrast, with increased spatial structure, the threshold becomes less clear. The village and subvillage scenarios required a post-MDA territory prevalence of 0.012% and 0.005%, respectively, for elimination in > 75% of simulations.

Similar threshold behaviour was observed for antigen prevalence (Additional file 4, Supplementary Fig. 10). In the territory scenario, LF was eliminated in most simulations when antigen prevalence was less than approximately 3% 1 year post-MDA. This threshold was reduced to approximately 1.3% and 0.8% in the village and subvillage scenarios, respectively. The high antigen prevalence relative to m.f. prevalence, seen in all simulations, was caused by the relatively large population of individuals with mature but non-breeding worms or residual antigen but no mature worms (Additional file 4 and Supplementary Fig. 11).

In spatially structured scenarios, if the threshold is analysed at the group-level instead of the territory-level, as the weighted mean of m.f. prevalence in groups with at least one m.f.-positive individual (Additional file 4, Supplementary Figs. 12 and 13), thresholds are increased. However, in the subvillage scenario, even after five rounds of MDA, a large number of endemic simulations still contained group(s) with notable m.f. prevalence.

### Surveillance

Following MDA, m.f.-positive individuals were found in small numbers of groups in the village and subvillage scenarios, with residual infections found in fewer groups in the subvillage scenario. Four rounds of MDA in the village scenario and five rounds of MDA in the subvillage scenario had similar probabilities of elimination (0.75 and 0.64). Despite the subvillage scenario containing almost five times the number of groups, residual infections were found 1 year post-MDA in 44% fewer groups in endemic simulations (Table 1). Similarly, in simulations where LF was eliminated, residual infections were found 1 year post-MDA in 70% fewer groups in the subvillage scenario compared with the village scenario.

**Table 1:** Mean number of groups with at least one m.f.-positive individual at 1 and 5 years post-MDA, sorted by intervention outcome for four rounds of MDA in the village scenario and five rounds of MDA in the subvillage scenario. Uncertainty is quantified with a 95% confidence interval

	Village		Subvillage	
	1 Year	5 Years	1 Year	5 Years
Endemic	3.37 (3.15–3.60)	1.62 (1.50–1.76)	1.95 (1.81–2.09)	1.61 (1.49–1.74)
Eliminated	2.33 (2.20–2.47)	0.43 (0.35–0.50)	0.69 (0.59–0.79)	0.16 (0.12–0.21)

**Fig. 6:**
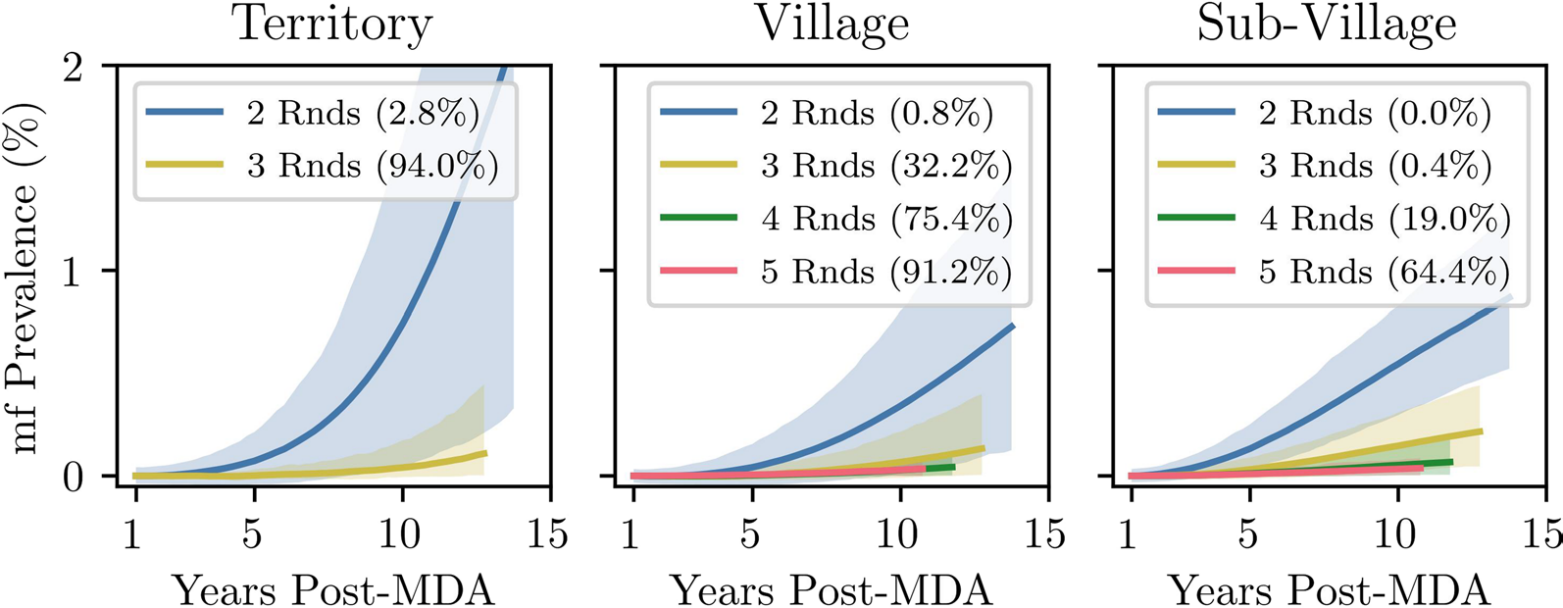
Mean increase in territory-level m.f. prevalence in the years post-MDA in endemic simulations. The percentage of simulations where LF was eliminated is given in brackets, and the shaded region is the 90% range

Where disease was not eliminated, there was limited spread in the years following MDA in the village and subvillage scenarios. Using the same example as above, residual infections were found in fewer groups 5 years post-MDA than 1 year post-MDA. Furthermore, scenarios with spatial structure exhibited a reduced rate of resurgence (Fig. 6). The rate of resurgence in the territory scenario with three rounds of MDA (94% elimination) was greater than that in the village and subvillage scenarios with either four or five rounds of MDA, despite the spatially structured scenarios having a lower proportion of simulations achieving elimination.

## Discussion

Heterogeneity is a key component of LF transmission, arising from the interplay of individual-level and spatial heterogeneities [19]. By varying the magnitude of spatial structure in a LF model, we were able to assess the importance of both types of heterogeneity in fitting to American Samoan survey data. In the territory scenario with no spatial structure, high levels of individual-level heterogeneity of mosquito bite exposure were required, and transmission was driven by a highly exposed and infectious subset of the population. With increasing levels of spatial structure, the fitted individual-level heterogeneity in bite risk decreased, and transmission was driven by a moderately infectious population in spatially focused areas.

Increased spatial structure led to an increase in the number of rounds of MDA required for elimination. Despite equal coverage, the highly spatial subvillage scenario required at least four more rounds of MDA than the non-spatial territory scenario to achieve comparable likelihoods of elimination. Furthermore, the territory scenario displayed a more evident elimination threshold following MDA. Below a critical number of rounds, LF remained endemic in nearly all simulations, while at or above the threshold, LF was eliminated in almost all simulations. This critical prevalence threshold in the territory scenario was substantially below the 1% m.f. threshold mandated by the WHO for the cessation of MDA and the commencement of TAS.

In the spatially structured scenarios, threshold behaviour was less evident. The probability of elimination increased gradually with additional rounds of MDA, and any threshold, if present, was lower than that observed in the homogeneous scenario. de Vos et al. [33] similarly found that, for onchocerciasis, increased spatial heterogeneity – modelled with assortative mixing – increased the stability of onchocerciasis at low prevalence and reduced intervention effectiveness.

Limitations in blanket prevalence thresholds for LF have been noted in several previous studies, which have found that the threshold for elimination may be significantly lower than 1% [10] and can be influenced by local conditions [9, 34, 35]. WHO guidelines specify thresholds to determine when MDA can cease; however, the implementation and evaluation units on which thresholds are calculated can have populations of up to two million [36]. In settings with highly focal transmission, threshold targets may not guarantee elimination where the evaluation unit size is large. Moreover, existing surveillance strategies may be unable to identify continued transmission in settings with heterogeneous transmission [37, 38]. As in Prada et al. [39], we found that in all scenarios, low levels of infection could persist for years post MDA. However, the introduction of spatial structure meant residual infections remained in a minority of groups, while resurgence was often slowed. This suggests that, particularly in a highly focal setting, LF could persist at low levels beyond the TAS timeline before there was a detectable resurgence.

American Samoa failed TAS-3 after previously passing TAS-1 and TAS-2 [7]. This failure, coupled with the predicted low post-MDA prevalence, spatially heterogeneous residual infections and potentially low thresholds, suggests the need for additional or alternative surveillance strategies. Groups with higher baseline prevalence have been observed to have an increased likelihood of residual infection post-MDA in Ghana [40] and Nepal [41]. These baseline surveys could be used to inform locations of post-intervention surveys. Furthermore, targeted surveillance could be an efficient and cost-effective strategy for detecting hotspots in large populations [42, 43]. Alternatively, molecular xenomonitoring (MX) has been able to detect residual hotspots post-MDA, find evidence of resurgence [44, 45] and may be more sensitive than surveys of antigen or m.f. in humans to detect pathogen signals in low prevalence settings [46]. MX could be especially insightful in spatially heterogeneous settings with limited vector dispersal. Diagnostic performance and cost-effectiveness are essential in assessing future surveillance strategies [47].

Understanding the spatial scale of LF transmission is critical due to its effects on transmission and surveillance. In American Samoa, post-MDA surveys have identified clustered residual transmission at the household level [13, 31], indicating that LF transmission is spatially heterogeneous and likely best captured by the highly spatially structured subvillage model. Several factors may influence the local spatial scale of LF transmission in American Samoa, including the limited dispersal of *Aedes polynesiensis* compared with other mosquito species [48, 49], the highly efficient limitation behaviour of the *Aedes* genus [27–29], the high relative population density and year-round high vector density [50].

Our study has several limitations. First, the model was only fitted to survey data from 2010 and 2016, which did not include data since American Samoa switched from two-drug to triple-drug MDA, and we assumed that the degree of clustering in the model was similar at these two time points. As the model was only fitted to surveys at low prevalence, the model may have limited accuracy at higher prevalence. Second, we modelled American Samoa as an isolated system; however, there is significant population mobility between the Samoan islands [51], which may allow for the importation of LF parasites [43]. Third, the model did not explicitly model m.f. density. Instead, we adopted a parsimonious model where an individual’s infectiousness was determined only by their mature mated worm load and the strength of limitation present in the model. As infectiousness was determined as soon as they had a mature pair, this probably overestimates initial infectiousness. The model also contained no explicit spatial heterogeneity in vector biting rates. In American Samoa, environmental factors, such as tree cover, positively correlate with antigen and antibody positivity [52], suggesting that biting rates are likely not homogeneous across the study setting. We also assumed that group connections were governed by a radiation model. The model would benefit from setting-specific commuting patterns as individual movement can affect disease spread and persistence [53]. Finally, as the aim of this study is to explore heterogeneity rather than evaluating different strategies, simplifications to interventions were made. We assumed there was no systematic non-compliance or non-access to MDA, but a 2019 survey in American Samoa found non-adherence of 35% in antigen-positive individuals and 22% in antigen-negative individuals [54]. Our assumptions around the efficacy of the drug regimen used in MDA are likely optimistic. We assumed that triple drug treatment of a m.f.-positive people resulted in either the death or permanent sterilisation of all adult worms in the host. In Samoa, individuals treated with triple drug MDA had complete clearance if given and taken at the correct dose [55]; however, in Fiji, m.f. clearance 1-year post-MDA in m.f.-positive individuals treated with triple drug MDA was only 64.9% [56].

## Conclusions

LF transmission is driven by individual-level and spatial heterogeneities. The scale of spatial structure included in a model affects the relative contribution of each, influencing transmission dynamics, critical threshold estimates and intervention and surveillance efficacy. Specifically, in American Samoa, models that assume contact is well mixed across the entire population underestimate the risk of resurgence following cessation of MDA. Setting-specific data on the local spatial scales of vectors and individuals are needed to accurately model LF transmission dynamics and to inform potential intervention and surveillance decisions.

## Supplementary Information

Additional file 1.

Additional file 2.

Additional file 3.

Additional file 4.

## Data Availability

Model used in the study is available here: https://github.com/callummshaw/NETFIL
