# Supplementary material for: The effects of variable spatial aggregation on lymphatic filariasis transmission
Source: Parasit Vectors. 2025 Jan 9;18:3. doi: 10.1186/s13071-024-06582-1 (PMC11716132; doi:10.1186/s13071-024-06582-1)
Supplement: Supplementary file 1 — Additional file 1. [file 13071_2024_6582_MOESM1_ESM.pdf]

Additional File 1: Model Initialisation

Text S1. Model Initialisation

To account for the over-dispersed distribution of worms in a population, we assumed that the initial worm distribution in each group would follow a negative binomial (NB) distribution with initial aggregation (shape) parameter k_0. From May [1], assuming an NB distribution where adult male and female worms are distributed together, then the following relationship is true

$$p_{mf} = 2 - p_A - 2^{1+k_0}(1-p_A)\left(1+(1-p_A)^{1/k_0}\right)^{-k_0}, \tag{1}$$

where p_A is the antigen prevalence, and p_{mf} is mf prevalence. The model was seeded in 2010 with a surveyed p_A of 3.2% [2]. The 2010 survey did not include mf, but we inferred p_{mf} by using the following relationship of p_A and p_{mf} in the years proceeding MDA outlined by Xu et al. [3]:

$$\frac{p_A}{p_{mf}} = 5.8464\mathrm{e}^{-0.153T} + 2.77, \tag{2}$$

where T is the number of years since the last round of MDA. This allowed for k_0 to be solved in equation 1. The mean initial worm load (μ) in the population was then:

$$\mu = \frac{k_0\left(1-(1-p_A)^{1/k_0}\right)}{(1-p_A)^{1/k_0}}. \tag{3}$$

The probability of an agent having n worms was given by:

$$P(N=n) = \left(1-\frac{\mu}{\mu+k_0}\right)^{k_0}\frac{1}{n!}\frac{\Gamma(k_0+n)}{\Gamma(k_0)}\left(\frac{\mu}{\mu+k_0}\right)^{n}, \tag{4}$$

where N is a NB random variable representing the number of worms an agent has.

An agent was initially antigen positive if a randomly generated number from a uniform real distribution on $[0,1]$, was less than the group-level antigen prevalence. If antigen positive, the probability the agent had n worms, where $n > 0$, was:

$$P(N=n \mid N>0) = \frac{P(N=n)}{P(N>0)}. \tag{5}$$

For all scenarios territory-wide antigen prevalence was 3.2%. For the Village and Sub-Village scenarios, to account for initial clustering, groups had varied initial antigen prevalence. We used previously published data for American Samoa, on the village-level intra-cluster ICC value for prevalence in 2016 [4]. As there are several conflicting definitions of ICC, to guarantee that our model was reproducing the correct degree of correlation we wanted to ensure that estimates of ICC from model simulations were calculated with a similar method to the source of our ICC estimates. The published ICC values we used had been calculated from a mixed-effect logistic regression model with fixed effects (e.g. age and sex) and random effects for village and household. In this framework, group-level (i.e. household or village level) prevalence is assumed to be logitnormally distributed. The formula used by Lau et al. [4] for calculating ICC depends only on σ, the s.d. of normal random effects, and is independent of the fixed effects. As we had other means of initialising prevalence by age and sex, we did not use the full logistic regression model used by Lau et al. [4]. Instead, to reproduce a comparable distribution of group-level prevalence, we drew from a logit-normal

distribution with the same σ and mean equal to the territory-wide prevalence in 2010. We validated this method with a test scenario with N_g groups and N_p individuals per group. The model was subsequently applied to spatially heterogeneous scenarios, where we accepted the initialisation only if the territory-wide antigen prevalence was within $\pm 0.1\%$ of the observed 2010 baseline prevalence.

Text S2. Biting Risks

An individual's bite risk (b) was modelled as a gamma random variable with shape parameter k and scale parameter $1/k$. For each scenario, a single shape parameter was fitted and used to generate all agent's bite risks. Agents seeded with either mature or immature worms were more likely to have greater bite risks. To implement this, bite risks for all N agents were generated and sorted. The top N_α were shuffled and assigned to the N_{mf} mf positive agents. Of the remaining $N - N_{mf}$ bite parameters, the top N_β were shuffled and assigned to the $N_{I/A}$ agents who were mf negative but had either immature or unmated worms. The remaining values were reshuffled and assigned to the remaining agents. In this study setting, $N_{mf} \approx 290$ and $N_{I/A} \approx 1500$. We assumed $N_\alpha = 1400$ and $N_\beta = 4200$.

Figure S1. Group-level Population Distributions

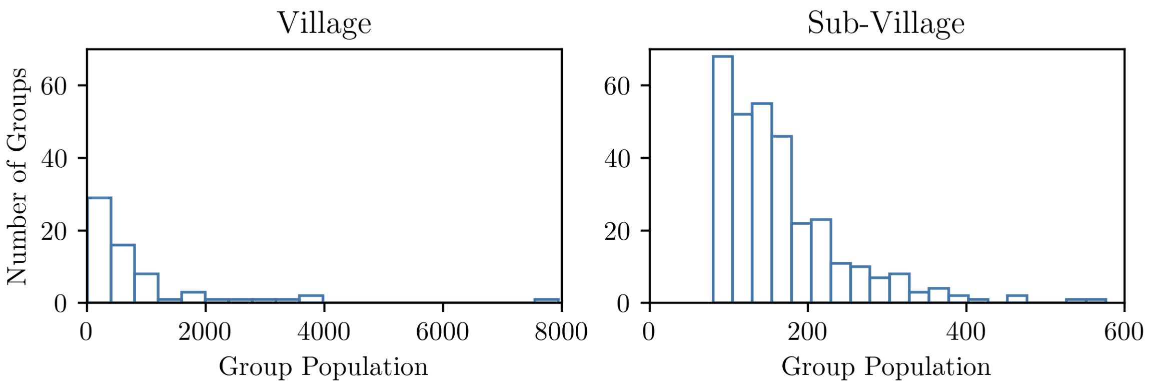

The group-level population distribution for the Village model is given in the left figure, and the Sub-Village model group-level population distribution is given in the right figure.

References

[1] May RM. Togetherness among schistosomes: its effects on the dynamics of the infection. Mathematical biosciences. 1977;35(3-4):301–343. https://doi.org/10.1016/0025-5564(77)90030-X.

[2] Lau CL, Won KY, Becker L, Magalhaes RJS, Fuimaono S, Melrose W, et al. Seroprevalence and spatial epidemiology of lymphatic filariasis in American Samoa after successful mass drug administration. PLoS Neglected Tropical Diseases. 2014;8(11):e3297. https://doi.org/10.1371/journal.pntd.0003297.

[3] Xu Z, Graves PM, Lau CL, Clements A, Geard N, Glass K. GEOFIL: A spatially-explicit agent-based modelling framework for predicting the long-term transmission dynamics of lymphatic filariasis in American Samoa. Epidemics. 2019;27:19–27. https://doi.org/10.1016/j.epidem.2018.12.003.

[4] Lau CL, Sheel M, Gass K, Fuimaono S, David MC, Won KY, et al. Potential strategies for strengthening surveillance of lymphatic filariasis in American Samoa after mass drug administration: Reducing 'number needed to test'by targeting older age groups, hotspots, and household members of infected persons. PLoS Neglected Tropical Diseases. 2020;14(12):e0008916. https://doi.org/10.1371/journal.pntd.0008916.
