# Supplementary material for: The effects of variable spatial aggregation on lymphatic filariasis transmission
Source: Parasit Vectors. 2025 Jan 9;18:3. doi: 10.1186/s13071-024-06582-1 (PMC11716132; doi:10.1186/s13071-024-06582-1)
Supplement: Supplementary file 2 — Additional file 2. [file 13071_2024_6582_MOESM2_ESM.pdf]

Additional File 2: MDA Assumptions

Table S1. MDA Assumptions

Variable	3D-MDA (IDA)
Probability of worm death (%)	55
Probability of full worm sterilisation (%)	45
Probability of partial worm sterilisation (%)	0
Duration of sterilisation (years)	Permanent

Triple drug MDA (3D-MDA) IDA assumptions.
