# Supplementary material for: The effects of variable spatial aggregation on lymphatic filariasis transmission
Source: Parasit Vectors. 2025 Jan 9;18:3. doi: 10.1186/s13071-024-06582-1 (PMC11716132; doi:10.1186/s13071-024-06582-1)
Supplement: Supplementary file 3 — Additional file 3. [file 13071_2024_6582_MOESM3_ESM.pdf]

Additional File 3: ABC Fitting Posteriors

Figure S2. Base Infectivity Posterior

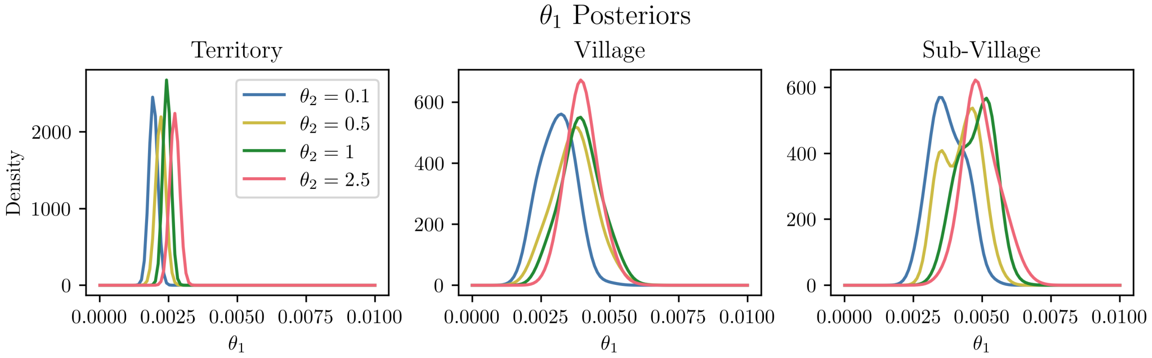

The posterior distributions for base infectivity (θ_1). Posteriors for Territory simulations are given in the left plot, Village posteriors in the centre plot, and Sub-Village posteriors in the right plot. In blue are simulations with $\theta_2 = 0.1$, in gold are simulations with $\theta_2 = 0.5$, in green are simulations with $\theta_2 = 1$, and in red are simulations with $\theta_2 = 2.5$.

Figure S3. Bite Aggregation Posterior

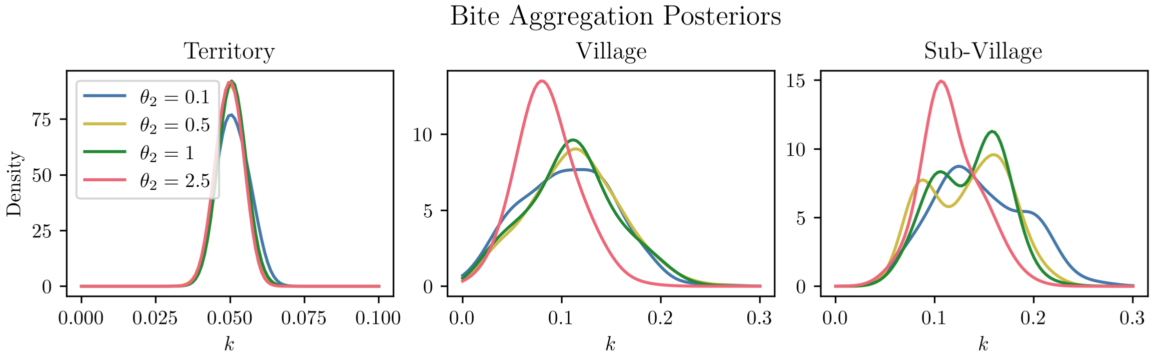

The posterior distributions for bite aggregation (k). Posteriors for Territory simulations are given in the left plot, Village posteriors in the centre plot, and Sub-Village posteriors in the right plot. In blue are simulations with $\theta_2 = 0.1$, in gold are simulations with $\theta_2 = 0.5$, in green are simulations with $\theta_2 = 1$, and in red are simulations with $\theta_2 = 2.5$.

Figure S4. Relative Bite Location Posterior

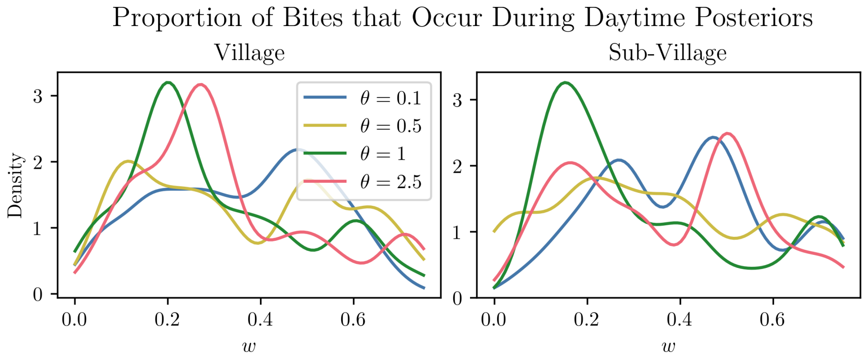

The posterior distributions for the ratio of day-time location bites to night-time location bites (w). Posteriors for Village simulations are given in the left plot, and Sub-Village posteriors are given in the right plot. In blue are simulations with $\theta_2 = 0.1$, in gold are simulations with $\theta_2 = 0.5$, in green are simulations with $\theta_2 = 1$, and in red are simulations with $\theta_2 = 2.5$.
