# Supplementary material for: The effects of variable spatial aggregation on lymphatic filariasis transmission
Source: Parasit Vectors. 2025 Jan 9;18:3. doi: 10.1186/s13071-024-06582-1 (PMC11716132; doi:10.1186/s13071-024-06582-1)
Supplement: Supplementary file 4 — Additional file 4. [file 13071_2024_6582_MOESM4_ESM.pdf]

Additional File 4: Full Model Results

Figure S5. Antigen and mf Prevalence Without MDA

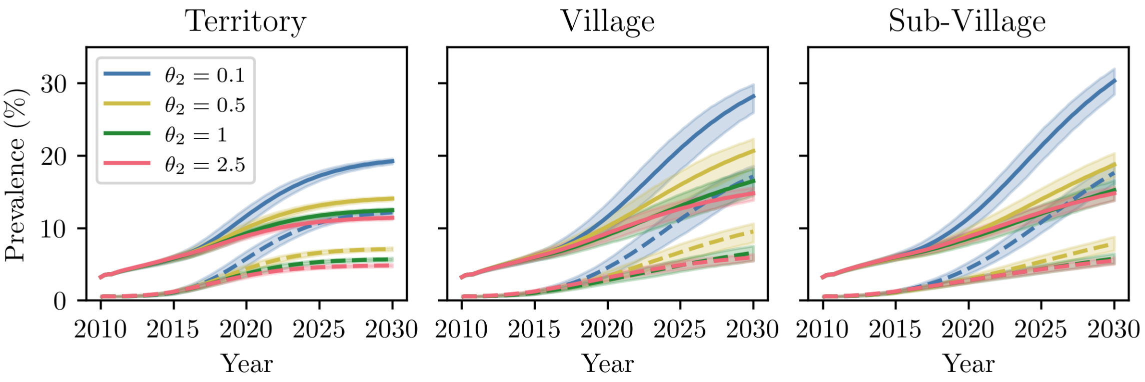

Mean territory-level LF prevalence for all tested levels of spatial aggregation and limitation strengths. Mean antigen prevalence is shown by a solid line, and mean mf prevalence is given by a dashed line. The shaded region is the 90% range.

Figure S6. Post-MDA Infection Distribution

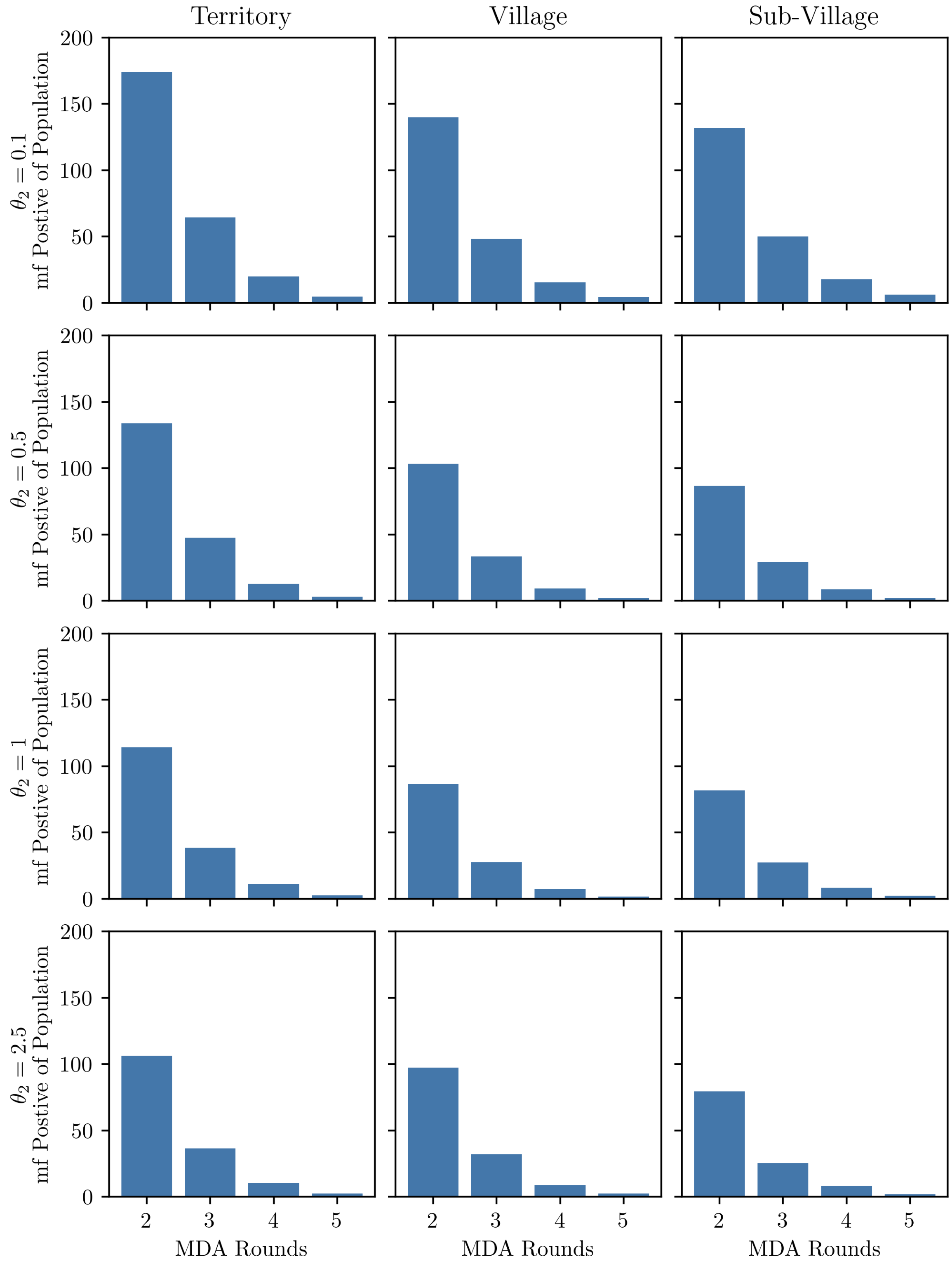

The distribution of mf positive infections one-year post-MDA for different MDA intervention lengths.

Figure S7. 70% MDA Effectiveness

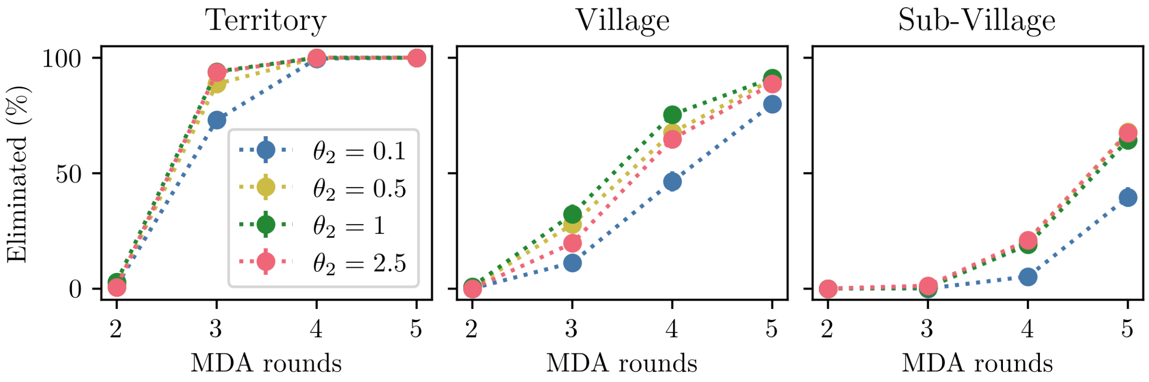

70% MDA coverage. Percentage of simulations where LF has been eliminated for 2-5 annual rounds of MDA for all tested levels of spatial aggregation and limitation strengths. 500 simulations per configuration. The error bars indicate 95% credible intervals for elimination.

Figure S8. 80% MDA Effectiveness

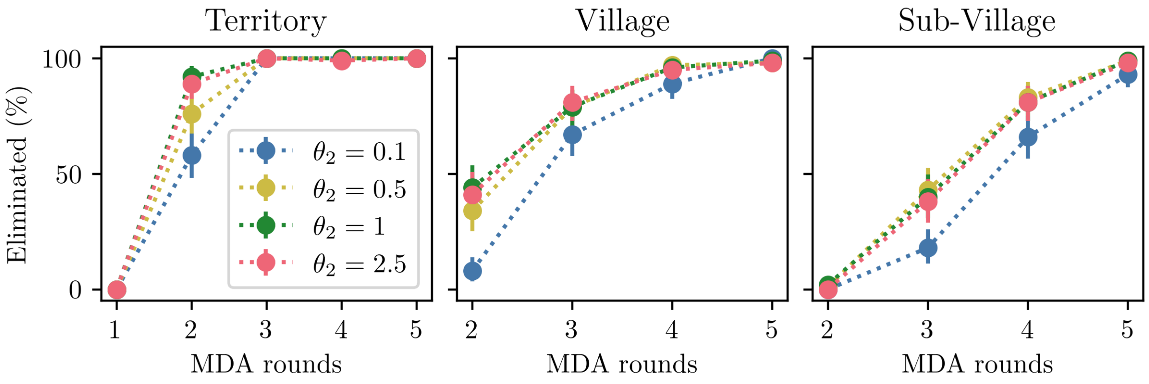

80% MDA coverage. Percentage of simulations where LF has been eliminated for 2-5 annual rounds of MDA for all tested levels of spatial aggregation and limitation strengths. 100 simulations per configuration. The error bars indicate 95% credible intervals for elimination.

Figure S9. Territory Level mf Threshold

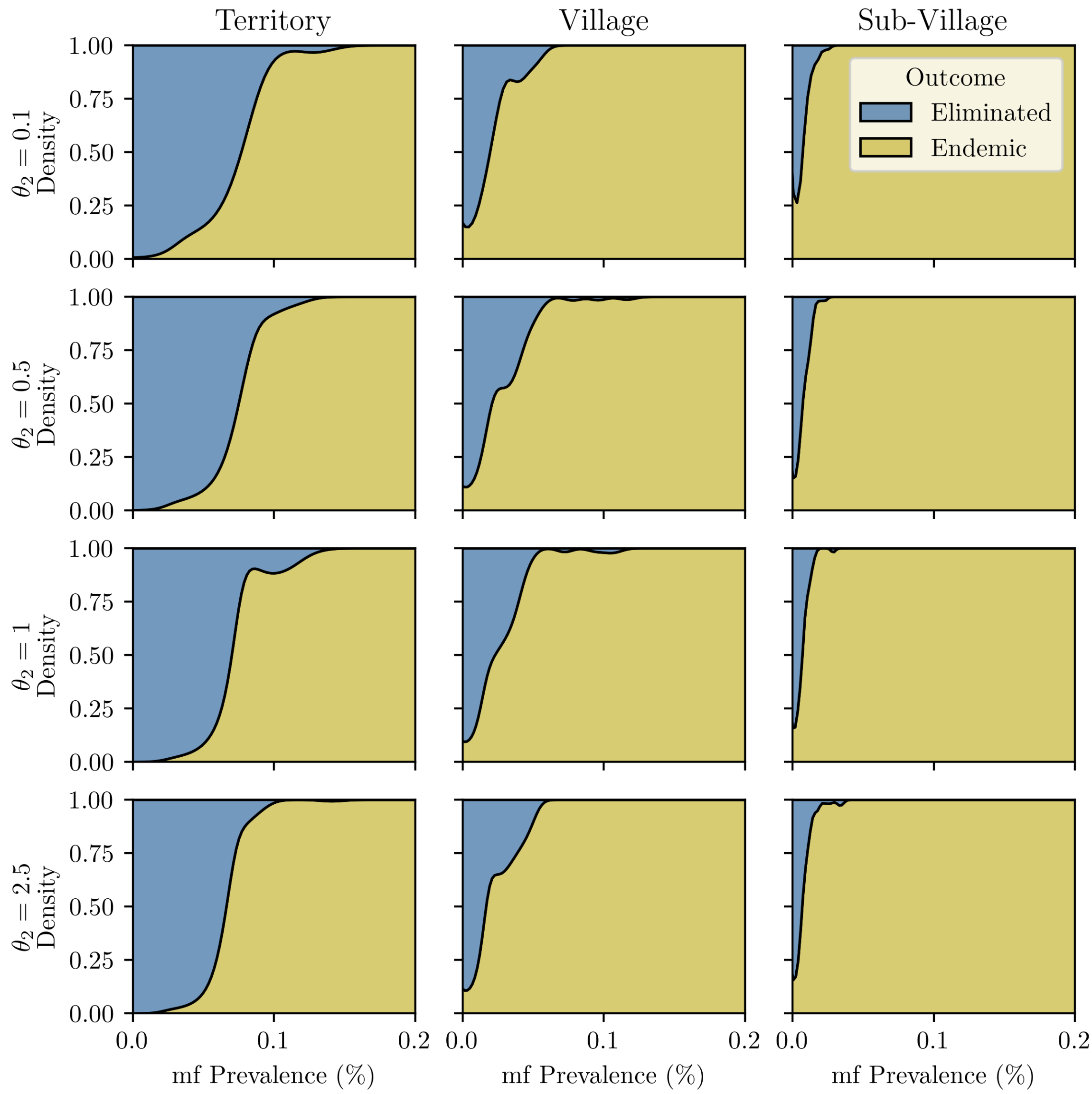

Probability of territory-wide elimination of LF, conditional on mf prevalence one year after the cessation of MDA. Simulations where LF remained endemic are in gold or was eliminated are in blue and each row corresponds to a different limitation strength. Prevalence is calculated for the three scenarios at the territory level.

Figure S10. Territory Level Antigen Threshold

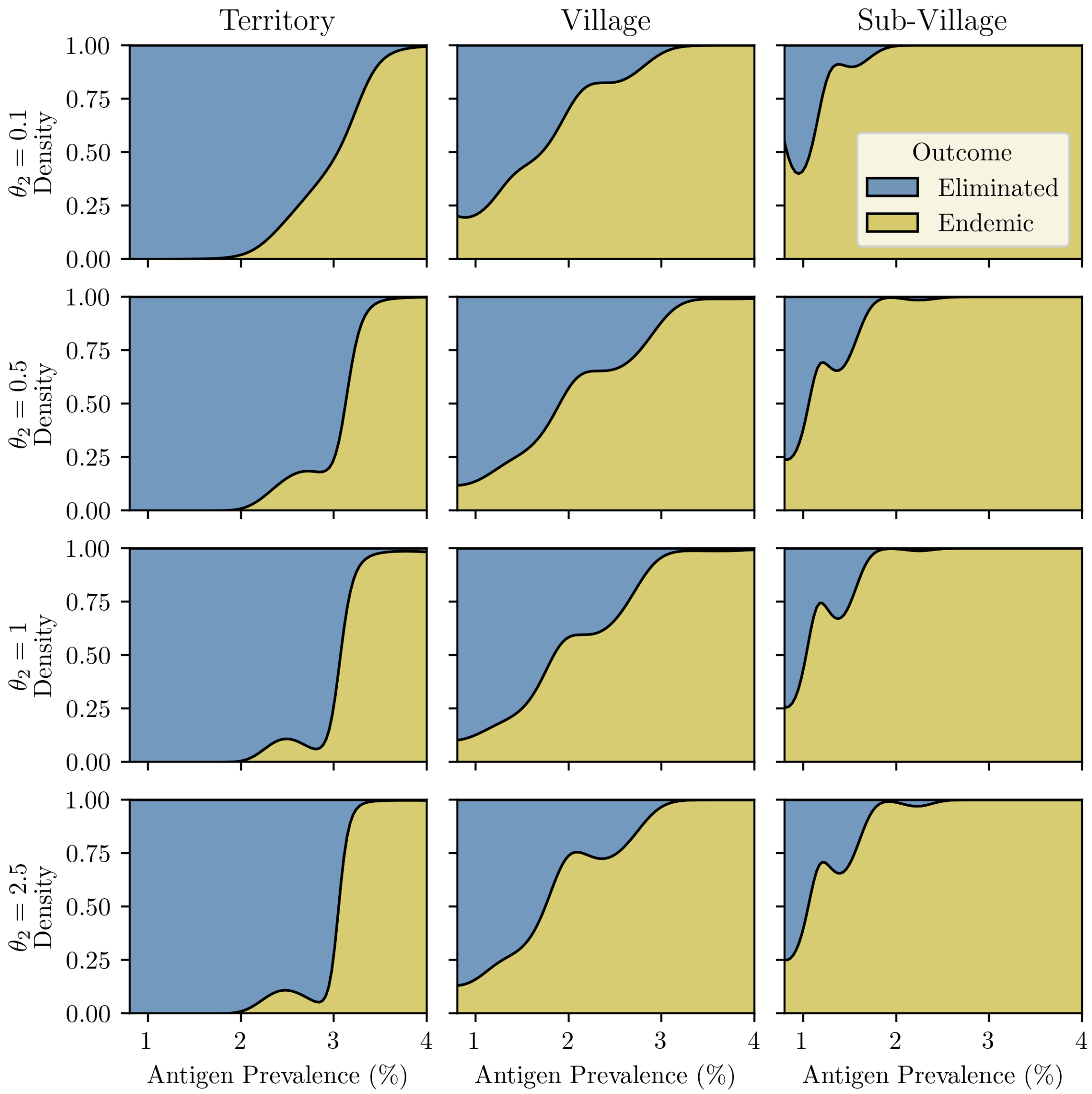

Probability of territory-wide elimination of LF, conditional on antigen prevalence one year after the cessation of MDA. Simulations where LF remained endemic are in gold or was eliminated are in blue and each row corresponds to a different limitation strength. Prevalence is calculated for the three scenarios at the territory level.

Figure S11. Post-MDA Infection Distribution by Infection Type

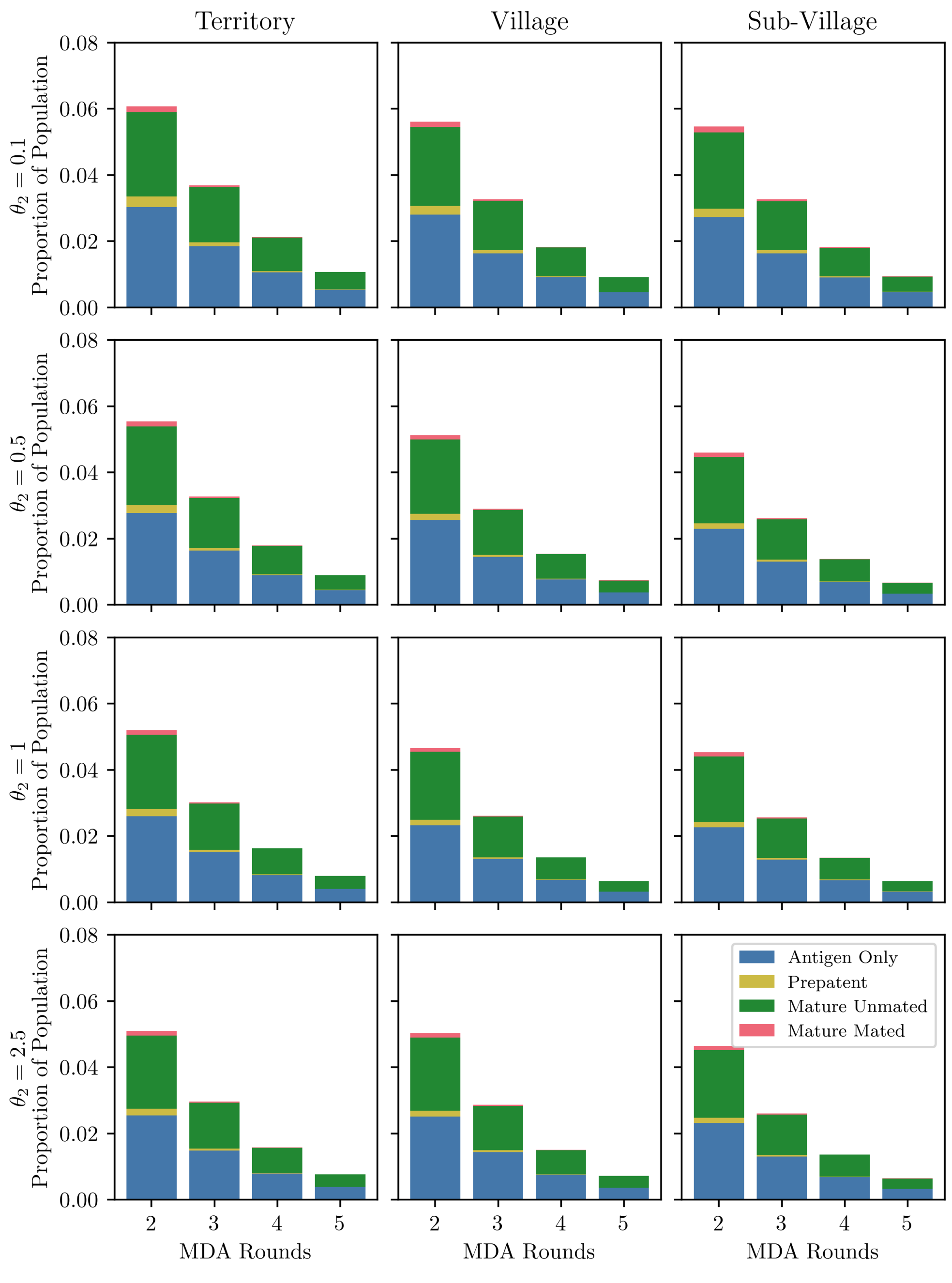

The distribution of LF infections one-year post-MDA for different MDA intervention lengths. In blue is the proportion of people who are antigen-positive but have no mature worms. In gold is the proportion of people with only prepatent worms, in green is the proportion of people with mature worms but not a breeding pair, and in coral is the proportion of people with at least one mated breeding pair of worms. Antigen-only positivity stems from the residual antigen that remains after the worms die. The residual antigen is estimated with a simple exponential decay model with an assumed half-life of 90 days.

Figure S12. Village Scenario Group Level mf Threshold

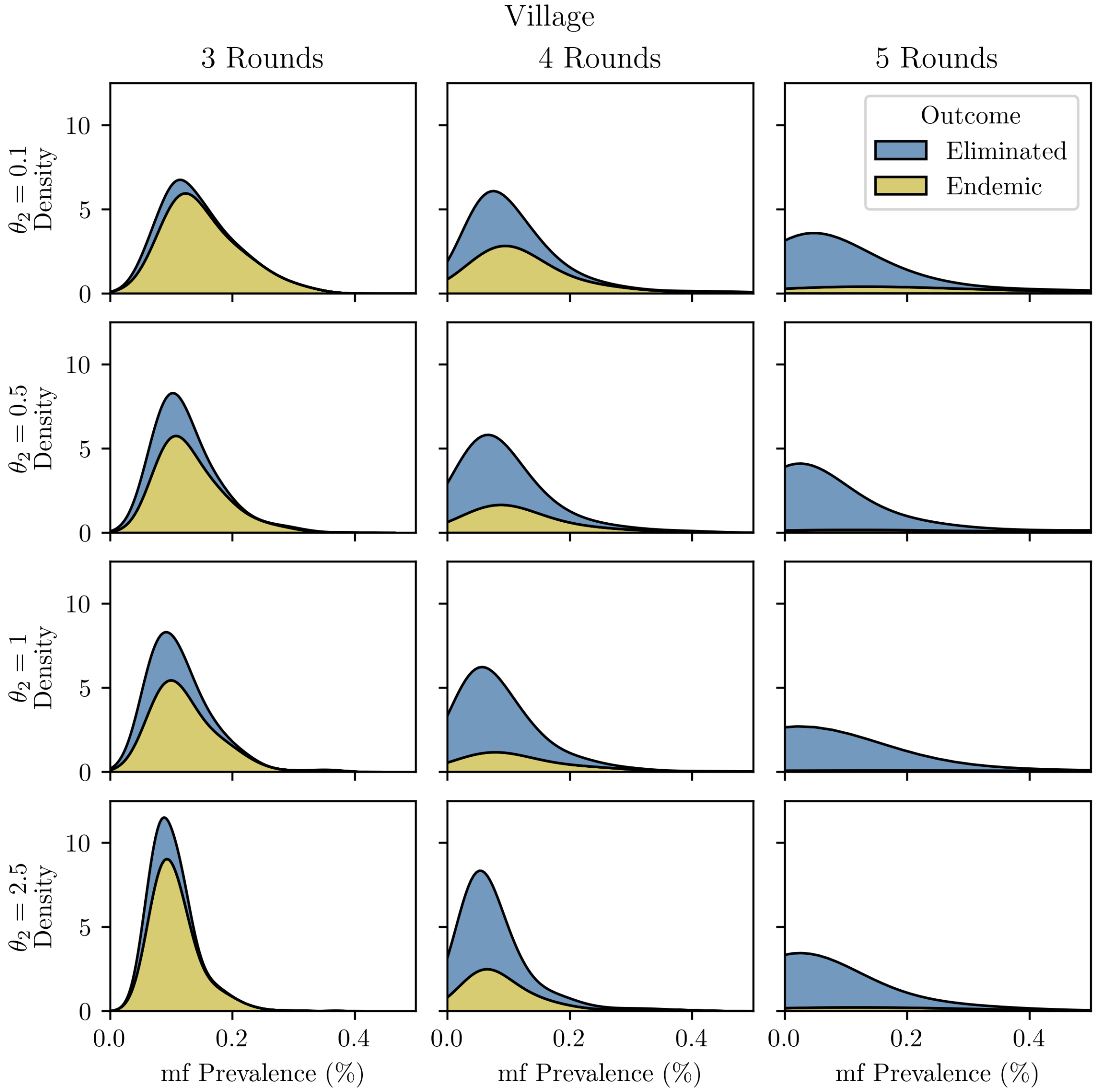

Kernel density estimate plots of group-level mf prevalence one year after the final round of MDA for the Village scenario, where LF remained endemic or was eliminated. Prevalence was calculated as the weighted mean of mf prevalence in groups with at least one mf-positive individual one year after MDA. Each column corresponds to the number of rounds of MDA implemented, and each row corresponds to a different limitation strength. Simulations where LF has been eliminated are given in blue, and endemic simulations are given in gold.

Figure S13. Sub-Village Scenario Group Level mf Threshold

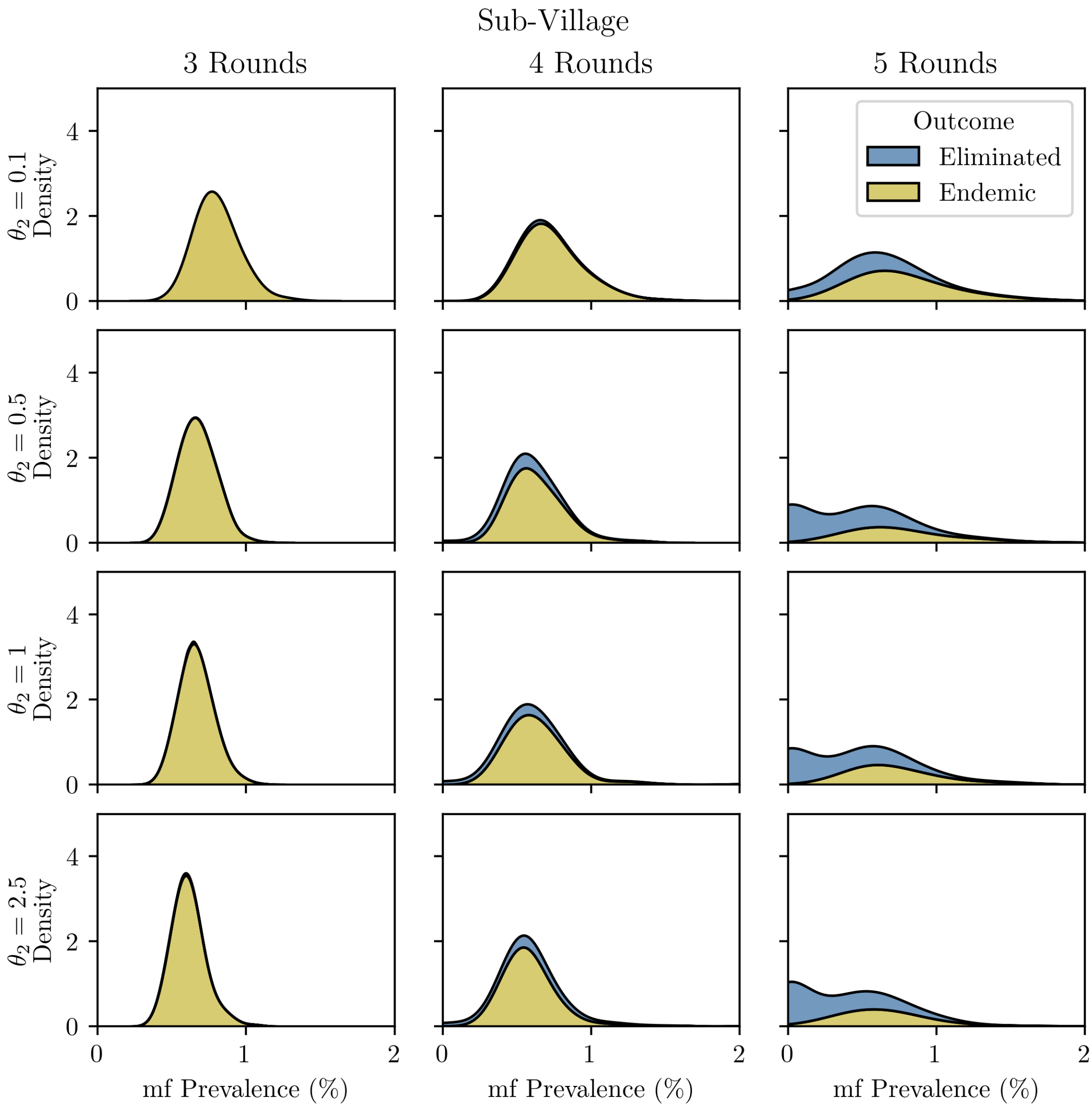

Kernel density estimate plots of group-level mf prevalence one year after the final round of MDA for the Sub-Village scenario, where LF remained endemic or was eliminated. Prevalence was calculated as the weighted mean of mf prevalence in groups with at least one mf-positive individual one year after MDA. Each column corresponds to the number of rounds of MDA implemented, and each row corresponds to a different limitation strength. Simulations where LF has been eliminated are given in blue, and endemic simulations are given in gold.

Figure S14. Village Scenario Residual Infection

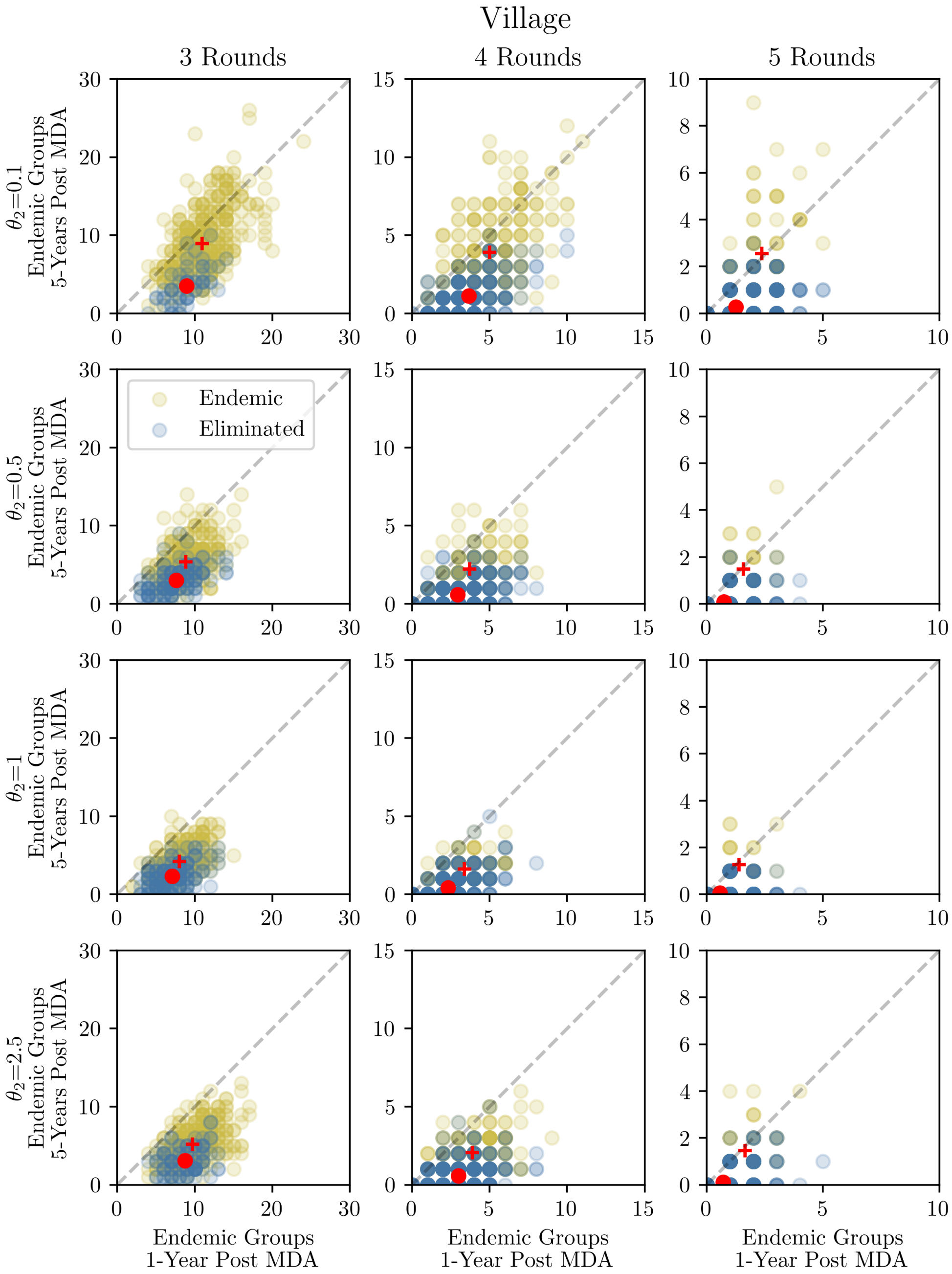

Village scenario: number groups with at least one mf-positive person one-year and five-years post MDA. Each dot represents a simulation; dots are coloured blue if LF was eliminated and are coloured gold if LF remained endemic. Each row corresponds to a different limitation strength, and each column has a different number of MDA rounds. The red cross is the mean value for endemic simulations, and the red circle is the mean value for elimination scenarios. The dashed black line is y=x.

Figure S15. Sub-Village Scenario Residual Infection

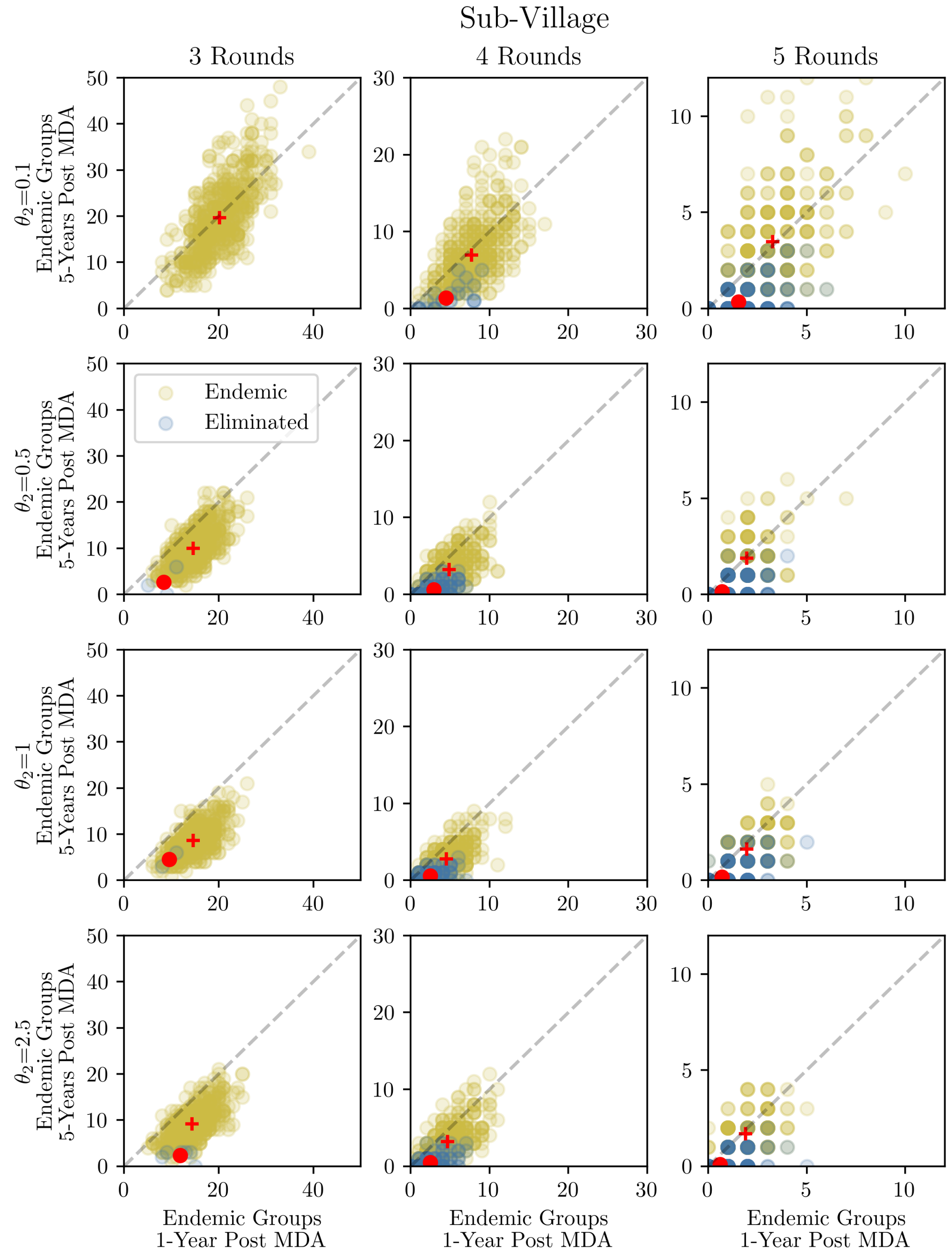

Sub-Village: number groups with at least one mf-positive person one-year and five-years post MDA. Each dot represents a simulation; dots are coloured blue if LF was eliminated and are coloured gold if LF remained endemic. Each row corresponds to a different limitation strength, and each column has a different number of MDA rounds. The red cross is the mean value for endemic simulations, and the red circle is the mean value for elimination scenarios. The dashed black line is y=x.

Figure S16. Rate of Resurgence

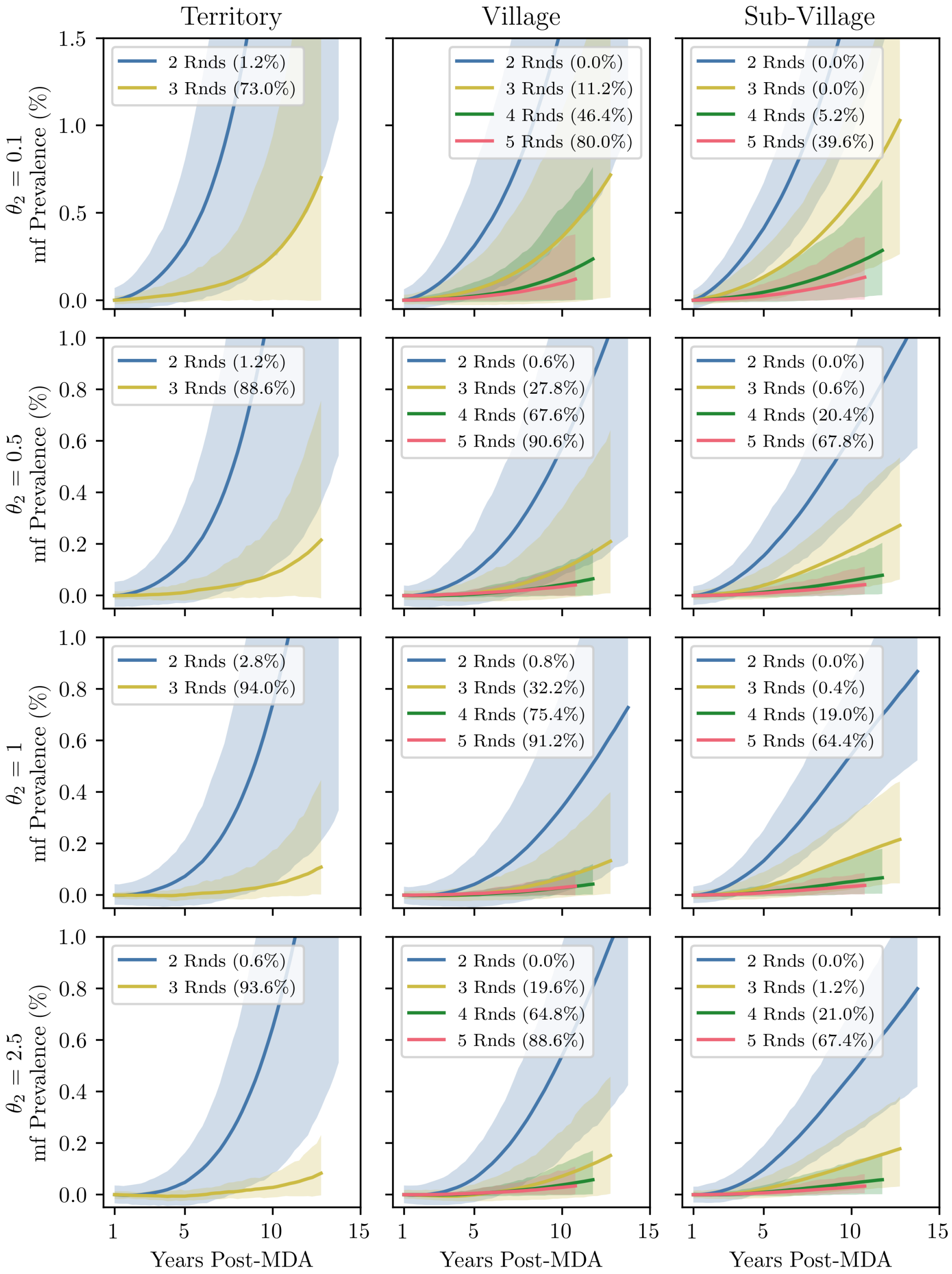

Mean increase in territory-level mf prevalence in the years proceeding MDA in endemic simulations. Each row corresponds to a different limitation strength, the percentage of simulations where LF was eliminated is given in brackets, and the shaded region is the 90% range.
